# Accuracy and functional outcomes of robot-assisted total knee arthroplasty combined with inverse kinematic alignment: a 500-case cohort study

**DOI:** 10.1051/sicotj/2026043

**Published:** 2026-07-28

**Authors:** Bryan Bertot, Arnaud Royet, Alban Stordeur, Frédéric Farizon, Pierre-Henri Vermorel, Rémi Philippot

**Affiliations:** CHU Saint-Étienne Avenue Albert Raimond 42270 Saint-Priest-en-Jarez France

**Keywords:** Arthroplasty, Replacement, Knee/methods, Robotic Surgical Procedures/methods, Knee Prosthesis

## Abstract

*Background:* Robot-assisted techniques combined with inverse kinematic alignment in knee arthroplasty allow for improved intraoperative management of lower limb alignment, particularly regarding the Hip–Knee–Ankle (HKA) angle. Postoperative HKA outliers, associated with mechanical and functional complications, may be reduced. Our study aims at determining the incidence rate of HKA outliers at 12 months postoperatively in patients who underwent Robot-Assisted Total Knee Arthroplasty (RA-TKA). Secondary objectives were to evaluate functional outcomes, postoperative HKA deviation, and complication rates at 12 months. *Methods:* We conducted a retrospective cohort study including all RA-TKA procedures performed in our center between 2020 and 2022. The arthroplasty technique was assisted by the Stryker MAKO™ robotic system combined with inverse kinematic alignment. Patients underwent clinical follow-up for 12 months, enabling the collection of radiological, clinical, and functional data. An HKA outlier was defined as a deviation greater than 3° at 12 months compared to the target HKA obtained immediately postoperatively, measured by the Stryker MAKO™ system. *Results:* The cohort included 500 arthroplasties, of which 487 provided complete data. The outlier rate obtained with RA-TKA was 2.05%. The mean difference between immediate postoperative HKA (HKA-D0) and HKA at 12 months (HKA-M12) was 1.3° (SD ± 1.2°). At follow-up, mean functional scores were 214 (SD ± 31) for KSS-2011 and 82 (SD ± 14) for KOOS. Mean range of flexion was 127° (SD ± 11°). Regarding safety outcomes, 1.2% of patients experienced sepsis, and 2.3% developed joint stiffness requiring mobilization under general anesthesia. *Conclusions:* This large cohort study demonstrates a low incidence of HKA outliers (only 2.05%) at 12 months postoperatively. Functional scores and range of motion indicate good recovery. Although this observational and non-comparative study does not allow definitive conclusions, the findings provide encouraging insights into alignment and functional outcomes following the combination of robot-assisted surgery and inverse kinematic alignment. *Level of Evidence:* III. Retrospective Cohort.

## Introduction

Total Knee Arthroplasty (TKA) is a common surgery performed on 1 million patients per year worldwide [1]. The dissatisfaction rate remains high, up to 30% of patients, depending on the series [2]. Outcomes depend on factors such as alignment strategy, implant positioning accuracy, implant design, and postoperative rehabilitation [2].

Knee arthroplasty aims to restore alignment of the lower limb at the Hip-Knee-Ankle (HKA) angle. Deviations greater than 3° from the intended postoperative value are considered outliers and may negatively affect mechanical and functional results [3]. Postoperative HKA depends on the precision of bone cuts and implant positioning.

Inverse kinematic alignment (iKA) is a TKA implantation technique designed to restore the pre-arthritic proximal tibial functional mechanical angle as closely as possible. Described in 2020 by P. Winnock de Grave et al., this involves a tibial cut respecting joint-line obliquity, followed by femoral adjustments to balance flexion and extension gaps, ensuring comparable residual laxity in both medial and lateral compartments [4]. This method requires providing a checked alignment, particularly in varus, in order to reduce the incidence of aseptic loosening, as suggested by Tueckin et al. [5].

Robot-assisted total knee arthroplasty (RA-TKA) surgery is a fast-growing field, offering modeling, navigation, and mechanical assistance, helping to improve the precision of bone cuts, implant positioning, and consequently the achievement of preoperatively defined alignment goals [6]. RA-TKA allows preservation of soft tissue and decreases intraoperative blood loss [7]. The Stryker MAKO™ Robot System (MAKO SmartRobotics™, Stryker Corporation, Kalamazoo, Michigan, USA), based on CT data, has been available since 2016 and has proven its effectiveness [8].

RA-TKA appears to improve radiological and functional results, compared with conventional TKA (C-TKA). Studies show a significant improvement in immediate post-operative radiological alignment with RA-TKA [9, 10]. A trend towards better functional scores also appears to be emerging [11, 12]. However, these studies contain numerous biases and do not allow us to formally settle on superiority.

The primary aim of our study is to evaluate the accuracy of the RA-TKA in achieving the iKA alignment goals defined pre-operatively through radiological assessment at 12 months post-operatively. Our secondary objectives are to evaluate clinical and functional outcomes and complication rates at 12 months post-operatively.

## Materials and methods

### Study design and study population

We conducted a single-center retrospective cohort study including all consecutive primary RA-TKA procedures performed between January 1, 2020, and December 31, 2022, using the MAKO Stryker™ robotic system combined with the inverse kinematic alignment technique at our university hospital. This comprises patients presenting with marked varus or valgus deformities, allowing for reflection on real-world clinical practice. Patients under 18 years or under legal protection were excluded.

### Surgical procedure

All interventions were performed using the MAKO Stryker™ robot-assisted surgery system by four experienced operators. We combined this with the inverse kinematic alignment (iKA) technique, similar to functional alignments, as described by P. Winnock de Grave, with the addition of a safety zone for the proximal mechanical angle of the tibial implant at 3° varus to 2° valgus [4, 5, 12–14]. The posterior-stabilized Stryker™ Triathlon implant was used.

The positioning of the femoral component was adjusted primarily by modifying its rotation to achieve identical spaces and residual ligament laxity at the internal and external compartments during both flexion and extension. This space balance was achieved using the navigation system of the MAKO Stryker™ system. A safety zone for the HKA angle was provided, ranging from 174° to 183°. The HKA angle was calculated preoperatively using the MAKO Stryker™ system, then adjusted intraoperatively.

### Data collection

At the end of the procedure, the HKA angle was measured using the MAKO Stryker™ system. This value (HKA-D0) corresponded to the final alignment obtained intraoperatively in the supine, non–weight-bearing position and was automatically calculated by the navigation system after bone cuts and implant positioning.

All patients were seen at 12-month follow-up. The HKA angle at 12 months (HKA-M12) was measured on a weight-bearing full-length standing hip–knee–ankle radiograph. Coronal alignment was measured using dedicated digital planning software on calibrated images, according to the mechanical axis definition (center of the femoral head to the center of the ankle joint). Range of motion, as well as the KSS-2011 and KOOS scores assessing physical abilities and residual functional limitations, were collected at the time of postoperative consultation [15, 16]. Patients who had not completed the functional outcome questionnaire were contacted by phone to obtain the missing data. Complications assessed included surgical site infection, capsular rupture, stiffness requiring mobilization under anesthesia, or arthrolysis.

Our primary outcome, the accuracy of implant positioning, was evaluated by the rate of outliers at 12 months postoperatively. Outliers were defined as a deviation of the HKA angle at 12 months strictly greater than 3° compared to the HKA angle measured intraoperatively, i.e., the absolute difference between the HKA-D0 and HKA-M12 angles as follows:

$$\text{DeltaHKA}=\left(\mathrm{HKA}-M12\right)—\left(\mathrm{HKA}-D0\right)$$

The secondary outcomes included:

- Mean difference between alignment planning and HKA measurement at 12 months.
- KOOS and KSS-2011 scores at 12 months.
- Knee range of motion in flexion at 12 months.

Safety outcomes analyzed were the incidence rate of the following postoperative complications:

- Surgical site infection.
- Non-septic incidents requiring surgical revision.
- Mobilization or arthrolysis under general anesthesia within 12 months postoperatively: maneuvers under anesthesia are indicated in cases of joint stiffness with a flexion of less than 90° on the 45th postoperative day.

### Statistical analysis

Statistical analyses were performed using Python 3 with Pandas, SciPy, NumPy [17]. Descriptive statistics were reported for all variables. Quantitative variables are expressed as means ± standard deviations, and qualitative data are presented as counts and percentages (%).

## Results

**Figure 1:**
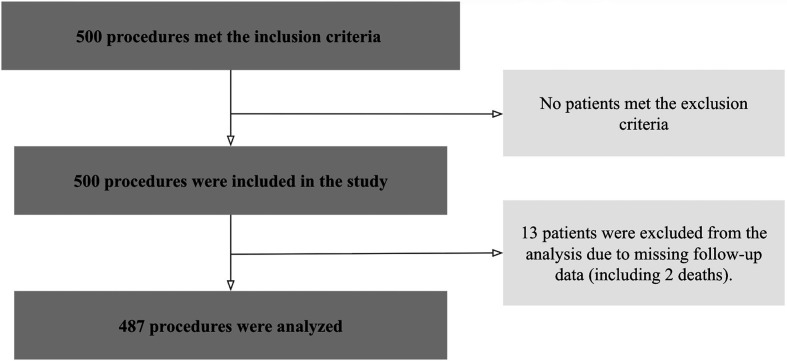
Flowchart.

From January 1, 2020, to December 31, 2022, a total of 500 arthroplasties were performed using the technique under investigation and were included in the cohort. Thirteen patients did not complete the 12-month postoperative radiographic follow-up and were excluded from the analysis (Figure 1). Patient characteristics are presented in Table 1.

**Table 1:** Preoperative characteristics of the study population.

Preoperative patient characteristics	Overall (*n* = 487)	Not-Outliers (*n* = 477)	Outliers (*n* = 10)
Age, years (Mean ± SD)	69.9 ± 8.2	69.9 ± 8.2	70.1 ± 7.8
Sex (M/F; %)	49.9% / 50.1%	50.3% / 49.7%	30% / 70%
Operated side (R/L; %)	54.4% / 45.6%	54.1% / 45.9%	60% / 40%
Type of deformity (Varus/Neutral/Valgus; %)	79.1% / 3.3% / 15.6%	80.7% / 3.4% / 15.9 %	100% / 0% / 0%
Preoperative frontal valgus deformity, ° (Mean ± SD)	184° ± 3	184 ° ± 3	
Preoperative frontal varus deformity, ° (Mean ± SD)	173° ± 4	174° ± 4	170° ± 4
Maximum preoperative flexion, ° (Mean ± SD)	125° ± 10	125° ± 10	124° ± 9

Analysis of the collected data revealed an outlier rate of 2.05% (*n* = 10/487) at 12 months postoperatively.

**Figure 2:**
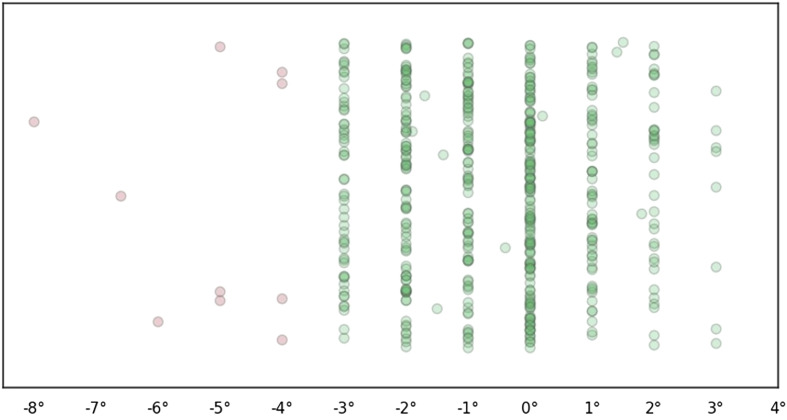
Results: Distribution of DeltaHKA.****DeltaHKA as defined: (HKA-M12) – (HKA-D0). ****1 point = 1 event. Outliers shown in red color.

The mean HKA deviation at 12 months was 1.3° (SD ± 1.2°). The distribution of the DeltaHKA angle deviation is illustrated in Figure 2. The functional scores obtained were 214/255 (SD ± 31) for the KSS-2011 and 82/100 (SD ± 14) for the KOOS. Functional outcome data were available for 335 and 333 patients for the KSS-2011 and KOOS, respectively, as some patients declined to complete the questionnaires during follow-up visits or telephone interviews. At 12 months postoperatively, the mean flexion range of motion was 127° (SD ± 11°). Detailed results are presented in Table 2.

**Table 2:** Results.

Postoperative follow-up results	Overall (*n* = 487)	Not-outliers (*n* = 477)	Outliers (*n* = 10)	*p*-Value (Welch’s T-test)
Outliers, % (*n*)	2.05% (10)			
|DeltaHKA|*,°(Mean ± SD)	1.3° ± 1.2			
Joint flexion at 12 months, ° (Mean ± SD)	127° ± 11	127° ± 11	122° ± 12	0.189
KSS-2011 score (Mean ± SD) (*n* = 335)	214 ± 31	213 ± 32	225 ± 18	0.108
KOOS score (Mean ± SD) (*n* = 333)	82 ± 14	83 ± 14	86 ± 8	0.290

*Expressed as absolute value.

Among the 10 outlier cases, 6 patients presented with major preoperative varus deformities greater than 10°, and 4 patients had obesity (BMI > 30 kg/m^2^). No corrective intervention related to coronal alignment was required during the first postoperative year. The only secondary procedure performed was one manipulation under general anesthesia for postoperative stiffness. Notably, 7 of the 10 outliers exhibited a valgus alignment at the 1-year follow-up, despite all having presented with a preoperative varus deformity. Subgroup analysis comparing outliers and non-outliers demonstrated no statistically significant differences in KSS-2011 scores (*p* = 0.108), KOOS scores (*p* = 0.290), or maximal flexion at one year (*p* = 0.189).

Twenty-three complications occurred, including 6 cases of sepsis and 11 cases of joint stiffness requiring surgical intervention. All adverse event data are presented in Table 3.

**Table 3:** Safety outcomes.

Adverse events	Overall (*n* = 487)	Not-outliers (*n* = 477)	Outliers (*n* = 10)
Surgical site infection, % (*n*)	1.2 % (6)	1.2 % (6)	0%
Major joint stiffness**, % (*n*)	2.3 % (11)	2.1 % (10)	10% (1)
Traumatic capsular rupture, % (*n*)	0.8 % (4)	0.8 % (4)	0%
Others***, % (*n*)	0.4 % (2)	0.4 % (2)	0%

**Joint stiffness with flexion <90° at postoperative day 45: indication for mobilization or arthrolysis under general anesthesia.

***Others: Intra-articular hematoma: 1, Traumatic patellar fracture: 1.

## Discussion

The primary aim was to assess RA-TKA accuracy in achieving preoperative alignment targets by analyzing 12-month HKA outliers. RA-TKA showed excellent implantation accuracy with a low outlier rate (2.05%) and minimal HKA deviation (mean DeltaHKA 1.3°).

The cohort demonstrates a very low rate of HKA outliers. The robot-assisted system reduces inaccuracies associated with conventional mechanical alignment techniques. The real-time navigation system allows continuous intraoperative monitoring of alignment and verification of final implant positioning, guaranteeing its fidelity to the intended plan. Ligament balancing is optimized, preventing overpressure in one compartment that could lead to prosthetic subsidence. Even within the ±3° range, postoperative HKA deviation averaged 1.3°, reflecting stable outcomes.

In the literature, outlier rates for C-TKA range from 13% to 38% [9, 10]. Our findings support RA-TKA use for improved postoperative alignment. Previous studies on this topic were affected by biases such as heterogeneous surgical protocols and limited statistical power. They reported a very low proportion of outliers for RA-TKA.

HKA outliers are associated with poorer mechanical and functional outcomes [3, 13]. Comparing functional and clinical scores between RA-TKA and C-TKA remains a key topic. We assessed recovery by assessing the KSS-2011 and KOOS scores, with total scores of 214/255 and 82/100, respectively, reflecting highly satisfactory functional outcomes at 12 months. Given the primarily radiographic focus of our study, preoperative functional outcome data were not collected, which prevented us from evaluating changes in functional outcomes between the preoperative and postoperative periods. Prior studies had heterogeneous assessments and rarely used the most recent KSS-2011 score [15].

A previous small-scale study investigated RA-TKA combined with iKA [12]. It reported encouraging functional scores but lacked statistical power. A meta-analysis by Zhang et al. indicates RA-TKA superiority in postoperative KSS scores, though heterogeneity in surgical protocols and robotic systems limits direct applicability [18]. Overall, RA-TKA appears to provide better functional outcomes, but evidence on the combined RA-TKA + iKA technique remains limited.

We observed an overcorrection toward valgus alignment in most outlier cases; this pattern may be related to the management of severe varus knees. In these cases, minimal medial tibial resection is performed. Implant fixation may therefore rely on a predominantly sclerotic bone surface within the medial compartment. Subsequent medial tibial settling could progressively modify coronal limb alignment and contribute to the development of a more valgus alignment than that achieved intraoperatively. However, this hypothesis remains speculative and could not be formally assessed in the present study. Although no significant difference in functional outcomes was observed at short-term follow-up between aligned cases and outliers, this finding does not preclude potential long-term implications. Deviation from the intended intraoperative coronal alignment target may alter load distribution across the knee joint and could influence implant fixation over time. Longer follow-up is therefore necessary to determine whether such alignment differences impact implant durability [19].

Postoperative complications were low: surgical site infection rate 1.2% at 1 year, comparable to 1.5–2% for C-TKA, suggesting non-inferiority [20–22]. Joint stiffness requiring intervention occurred in 2.3% of cases; prior reports for C-TKA range from 2.5 to 4.8%, and less than 2% for RA-TKA [23, 24]. The improvement observed in the RA-TKA group may be explained by more precise intraoperative assessment of ligamentous laxity as well as guided bone preparation, allowing the achievement of patient-specific implant positioning targets. The combination of iKA and MAKO™ robotic navigation facilitates satisfactory ligament balancing, proper joint balance, and peripheral isometric deformation both internally and externally.

This study has several limitations. Although the 12-month follow-up provides relevant short-term radiographic and functional data, it remains shorter than the 2-year follow-up commonly reported in arthroplasty studies and is limited compared with the expected lifespan of a knee implant. While sufficient to assess early outcomes, this follow-up duration may not capture longer-term phenomena such as alignment drift, implant loosening, late complications, or the evolution and stabilization of functional outcomes beyond the first postoperative year. Early mechanical and functional improvements may persist over time, but this remains to be demonstrated. Longer-term follow-up investigations are currently underway at our institution and are expected to provide further insight into the durability of the radiographic and clinical outcomes.

The comparison between intraoperative and 12-month HKA values must be interpreted cautiously. Intraoperative alignment was recorded in the supine, non–weight-bearing position using the navigation system, whereas the 12-month measurements were obtained from standing weight-bearing radiographs. A systematic discrepancy between intraoperative MAKO™ measurements and postoperative weight-bearing radiographs may partly explain our findings. Glowalla et al. reported that postoperative full-leg standing radiographs consistently demonstrated less residual varus alignment than intraoperative MAKO™ measurements, with a mean difference of 1.8° between the two assessment methods, which could bias our findings [25]. These findings suggest that the robotic navigation system may tend to overestimate residual varus deformity intraoperatively. However, unlike Glowalla et al., our study did not include paired intraoperative and postoperative alignment measurements, preventing us from directly assessing the presence and magnitude of such a systematic measurement bias in our cohort.

As this study was observational, postoperative imaging followed the institutional standard protocol. This protocol does not include systematic early postoperative weight-bearing radiographs (day 1–2) or postoperative CT scans. Such additional imaging could have enabled comparison with the intraoperative measurement. However, it was not performed as it would have implied additional radiation exposure and increased cost beyond routine clinical care.

The study cohort is single-center, which may limit external validity. Finally, this study is observational and non-comparative; it does not allow definitive conclusions regarding the superiority of RA-TKA combined with iKA over C-TKA.

On the other hand, our cohort presents significant advantages. The study population is large, encompassing several hundred arthroplasties performed using a homogeneous protocol, a number rarely reached in the current literature [9, 10]. The surgical team performing the procedures consisted of experts, and the MAKO Stryker™ robot used has demonstrated efficacy and reliability. RA-TKA is rapidly expanding, and inverse kinematic alignment shows encouraging results. The combination of these two techniques appears to be a promising approach for TKA. To our knowledge, no study of this scale has investigated their combined use.

To conclude, RA-TKA combined with iKA enables the achievement of preoperatively defined alignment targets with excellent precision and a low 12-month outlier rate. These high-quality observational data highlight postoperative functional scores and joint ranges of motion, reflecting good recovery of the operated limb at 12 months. The alignment findings appear favorable in the context of published data, although the present study does not permit direct comparison.

## Data Availability

Data associated with this article cannot be disclosed due to legal reasons.
